# Integrating stages of change models to cast new vision on interventions to improve global retinoblastoma and childhood cancer outcomes

**DOI:** 10.1186/1471-2458-14-944

**Published:** 2014-09-11

**Authors:** Meaghann S Weaver, Christina L Heminger, Catherine G Lam

**Affiliations:** Department of Oncology, St. Jude Children’s Research Hospital, 262 Danny Thomas Place, Memphis, Tennessee 38105 USA; Milken Institute School of Public Health, The George Washington University, 2175 K Street NW, Washington, District of Columbia 20037 USA; International Outreach Program, St. Jude Children’s Research Hospital, 262 Danny Thomas Place, MS 721, Memphis, TN 38105 USA

**Keywords:** Retinoblastoma, Pediatric oncology, Stages of change model, Resource-limited settings, Interventions, Diagnostic barriers, Treatment barriers, Social efficacy

## Abstract

**Background:**

Retinoblastoma, the most common intraocular tumor globally, represents a curable cancer when diagnosed early and treated promptly. Delay to diagnosis, lag time prior to treatment initiation, and abandonment of treatment including upfront treatment refusal, represent stark causes of high retinoblastoma mortality rates in low- and middle- income settings, particularly regions in Africa. While a health delivery-based approach has been a historic focus of retinoblastoma treatments globally and is essential to quality care, this is necessary but not adequate. Retinoblastoma is a compelling disease model to illustrate the potential insights afforded in theory-informed approaches to improve outcomes that integrate public health and oncology perspectives, prioritizing both health service delivery and social efficacy for cure.

**Discussion:**

Given that barriers to appropriate and timely diagnosis and treatment represent main contributors to mortality in children with retinoblastoma in resource-limited settings such as certain areas in Africa, an important priority is to overcome barriers to cure that may be predominantly socially influenced, alongside health delivery-based improvements. While Stages of Change models have been effectively utilized in cancer screening programs within settings of economic and cultural barriers, this application of health behavior theory has been limited to cancer screening rather than a comprehensive framework for treatment completion. Using retinoblastoma as a case example, we propose applying stage-based intervention models in critical stages of care, such as the Precaution Adoption Process Model to decrease delay to diagnosis and a Transtheoretical Model to increase treatment completion rates in resource-limited settings.

**Summary:**

Stage-based theories recognize that improved cure and survival outcomes will require supportive strategies to progress households, communities, and social and economic institutions from being unaware and unengaged to committed and sustained in their respective roles. Applying a stage-based model lens to programmatic interventions in resource-limited settings has potential for visible improvement in outcomes for children with retinoblastoma and other cancers.

## Background

### Global priority based on incidence

Retinoblastoma represents the most common pediatric intraocular cancer with the greatest disease burden in populations with high birth rates. Across the globe, there are 9,000 new diagnoses of retinoblastoma estimated annually, or approximately one case per 15000 live births, with the majority in resource-limited settings [1]. Hospital- based studies in India suggest that this intraocular cancer may comprise up to 10-15% of all diagnosed childhood cancers in parts of India, in contrast to most developed settings where it accounts for less than 5% of pediatric cancers [2]. The varying reported incidence of retinoblastoma within countries likely reflects reporting mechanisms more than true incidence difference [3, 4]. Retinoblastoma has been reported as the most frequent solid tumor diagnosis in Mexico after central nervous system neoplasms [5] and in northern Nigeria, retinoblastoma is documented as the most common childhood malignancy overall, accounting for almost one-third of all pediatric oncology cases [6]. Retinoblastoma has been documented among the top three childhood cancers diagnosed in Tanzania, Ghana, the Congo, and Kenya and as the fourth most common in Senegal [7–11]. The documented recognition of retinoblastoma in low- and middle-income settings (LMIS) is believed to under-represent the actual number of cases [9, 12].

Among areas with lower overall detection of childhood cancers, there is an apparent high incidence in advanced stage retinoblastoma being reported [13]. This eye tumor progresses through signs that can be relatively subtle, such as strabismus (abnormal eye alignment often resulting in a squint), to leukocoria (white light reflex, which can be readily recognized by alert family and community members), to more advanced orbital inflammation, proptosis (orbital bulge) and bulky protruding extraocular masses literally staring at an observer both in community and clinical settings, accompanied by distant disease spread [14]. Retinoblastoma may therefore be more amenable to reporting in low- and middle-income settings globally as compared with other childhood oncologic diagnoses in these same settings that may be more heavily resource- or technology-dependent.

### Extent and impact of delay

**Figure 1:**
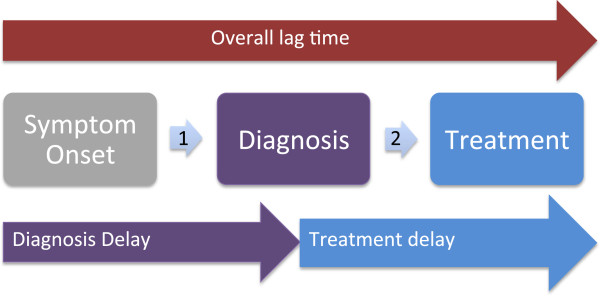
**Delineating lag time.** Delineating total lag time (red) prior to initiation of curative therapy as composed of delay to diagnosis (purple) and delay to treatment initiation (blue).

**Figure 2:**
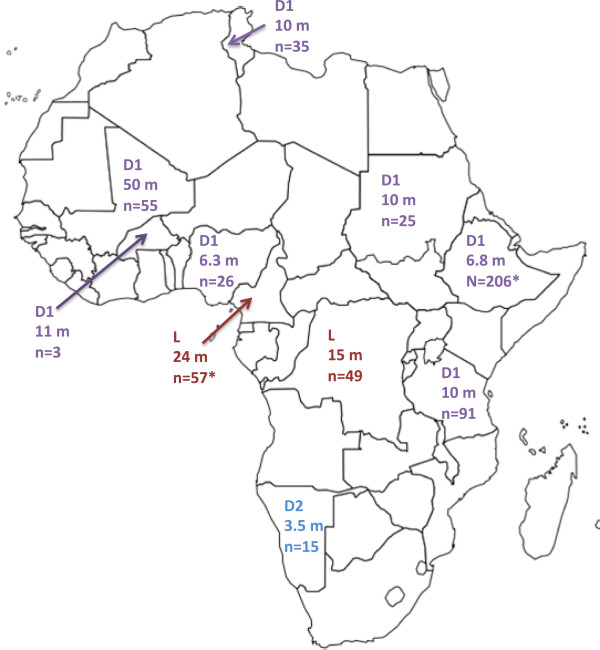
**Duration (in months) of delays to retinoblastoma treatment in Africa.** L = Delay from symptom to treatment, D1 = Delay from symptom onset to diagnosis, D2 = Delay from diagnosis to treatment initiation. * = month duration obtained through personal communication with authors and included with authors’ kind permission (unpublished data). Cameroon L = 24 months, n = 57 (Kagmeni2013*) [23]. Congo L = 24 months, n = 49 (Lukusa2012) [9]. Nigeria D1 = 6.3 months, n = 26 (Bekibele2009) [24]. Tanzania D1 = 10 months, n = 91 (Bowman2008) [17]. Mali D1 = 50 months, n = 55 (Boubacar2010) [18]. Tunisia D1 = 10 months, n = 35 (Frikha2009) [16]. Namibia D2 = 3.5 months, n = 15 (Wessels1996) [25]. Sudan D1 = 10 months, n = 25 (Ali2011) [26]. Burkina Faso, D1 = 11 months, n = 3 (Nikiema2009) [27]. Kenya D1 = 6.8 months, n = 206 (Nyamori2012*) [20].

**Figure 3:**
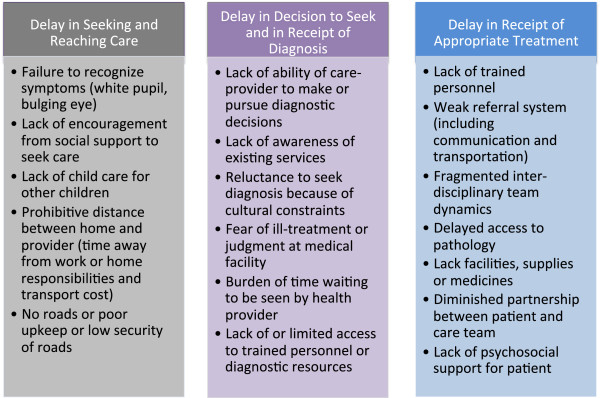
**Tri-lineage model of delay with description of possible causation.**

Retinoblastoma represents a curable cancer when caught early. Whether the lag time is due to delay in diagnosis or due to delay in treatment initiation (Figure 1), population-based studies reveal extended delays in access to cure in many LMIS. As an example, we explored quantified pediatric retinoblastoma lag times as reported in the continent of Africa through a comprehensive literature review and direct author contact for additional information regarding both published and unpublished data, with data available for 10 countries detailed (Figure 2). Mean time from symptom onset to diagnosis was reported by retrospective chart reviews as 6 months in Nigeria (n = 26) [15], 10 months in Tunisia (n = 35) [16], 8 months in Tanzania (n = 91) [17], and over 24 months in Mali (n = 50) [18]. A delay of more than six months from the first clinical sign of retinoblastoma to diagnosis is associated with assured extraocular spread and 70% mortality [3]. Of the cases of retinoblastoma diagnosed over a five-year period in central Africa, over 90% of the patients presented to the retinoblastoma center after extraocular spread [19]. In Kenya, where the delay from retinoblastoma symptom onset to diagnosis is a mean of 6.8 months, the mortality of retinoblastoma is 73% [20], which is over 50 times higher than that in Canada (1%) [21]. Approximately half to three-fourths of children diagnosed with retinoblastoma in Africa die, presumably due to diagnosis at an advanced stage, while 3-5% of children with retinoblastoma die in the United States and Europe, presumably in part due to earlier diagnosis alongside comprehensive management [3]. While current treatment emphasis in high-income countries focuses on vision-sparing interventions, the priority in LMIS remains the life-saving interventions of prompt diagnosis and treatment. A tri-modal depiction of delay (Figure 3) acknowledges that a conglomeration of health service delivery and social efficacy promotion must be established in the steps from first symptom recognition to diagnosis to urgent treatment initiation [22].

Medical factors interplay with complex and shifting social dynamics. As health delivery systems improve and as medical care advances, the management of retinoblastoma shifts from the basic priority of saving the life of the child with retinoblastoma, to include prioritization of ocular salvage. While the Stages of Change model we detailed here emphasizes early recognition and acceptance of enucleation (a survival priority) that may best apply to late presenting retinoblastoma in resource-limited settings, components and applications of this model may be adapted for other heterogeneous contexts. For instance, in contexts with earlier presentations and additional resources, consideration of the best interests of the child with intraocular disease may warrant weighing the continuum of readiness for intra-arterial or systemic therapy (an ocular salvage priority), and recognizing the perceptions and barriers that may affect uptake of these therapies where appropriate and available. Rather than view theory as a rule equally applicable in all settings, informed application of theory recognizes the unique and complex dimensions of the social and medical decisional continuums.

### Barriers to diagnosis

Health service delivery barriers to earlier diagnosis in LMIS include lack of universal health insurance for non-communicable diseases, missing links in multidisciplinary inclusion, constraints in procurements of diagnostic supplies, and inefficiencies within the referral system [24]. Local community health workers or general care providers may not have the referring mechanism readily in place to expedite a family’s access to diagnosis. Social barriers to receipt of a diagnosis include a myriad of educational, cultural, and economic influences. Lack of parental education and low awareness of the signs and symptoms of retinoblastoma can delay families from seeking medical attention [28]. Parents may fear judgment from the medical team if the retinoblastoma symptoms have progressed to the point of disfiguration prior to presentation to care. Even when a family does recognize the signs of retinoblastoma, some families may believe eye cancer is incurable and therefore select not to seek medical care (cancer fatalism) [10]. Chart assessments of causes for delay to retinoblastoma diagnosis in LMIS describe parental reliance on traditional healers or prayer camps for initial treatment due to available access and cultural prioritization [9, 29, 30], as belief-system has clear impact on care seeking [31, 32]. Conflicting priorities, to include survival priorities such as food or school fees for other siblings, may realistically serve as barriers to pursuit of a diagnosis. Parents may not be able to fund diagnostic procedures. Parents may also not have means of transport to medical centers due to prohibitive travel time, transportation fees, or lack of reliable or safe roads. Regional conflict and political strife risk disrupting vital health services and supply chains, which urges attentiveness to the needs of children with chronic conditions during times of acute conflict [33].

### Barriers to treatment initiation and completion

Despite receipt of a retinoblastoma diagnosis, the child’s life is jeopardized if necessary treatment is delayed [34]. Health service delivery barriers to earlier treatment initiation in LMIS include lack of cost coverage for curative-directed treatments; shortage of personnel; fragmented or delayed hand-offs between multi-disciplinary team members such as general providers, pathologists, oncologists, surgeons; constraints in access to surgical supplies; and medication shortages [24]. Even if the health delivery system was fluid and efficient, the cultural context of partnership, education, and enablement between families and providers remains critical. A family’s understanding of the treatment plan and trust in the health team remains a vital component to treatment efficacy and psychosocial wellness [35]. Refusal of enucleation (removal of the eye globe) has been documented as a main obstacle to cure in up to 40% of all patients diagnosed with retinoblastoma in low-income settings [36]. Families in some LMIS may reject enucleation as curative treatment because of actual or perceived social stigma or poor understanding of the high quality of life possible after unilateral enucleation [4]. A retrospective review of enucleation refusal from Nigeria reported >10% (3/26) upfront enucleation refusal rate and 42% abandonment rate prior to enculeation (11/23) [24]. Investigation of attitudes regarding blindness in Africa reveal continued prevalence of stigma attached to eye loss, particularly in rural communities [37, 38]. Furthermore, delays during treatment and treatment abandonment may adversely affect a dominant proportion of patients in resource-limited settings [14, 34, 39]. Ultimately, delays in diagnosis and treatment that result in more advanced, extraocular disease are unfortunately associated with requirements for more intensive therapy and expertise with greater demand for resources, higher morbidity and less chance of cure and survival. Children diagnosed early with disease well-confined within the eye, however, can often be saved with simpler surgical enucleation alone, thus addressing barriers to timely treatment have great potential to save resources as well as patients’ lives.

### Purpose statement

Viewing pediatric oncology interventions, such as retinoblastoma diagnosis and treatment interventions, through a comprehensive public health lens provides opportunity for new insights. A public health model (Table 1) can be applied to guide all programmatic steps from defining the problem through to intervention planning, implementation, and evaluation [40]:

**Table 1:** **Public health approach to retinoblastoma**

Programmatic phase	Public health approach
Definition phase	● Survey community awareness regarding retinoblastoma [41]
	● Monitor baseline knowledge of healthcare providers [5]
	● Assess attitudes regarding cancer susceptibility and severity
	● Determine extent of blindness stigma within community
	● Assess available local resources
	● Determine barriers to patient and provider efficacy
Planning phase	● Prioritize partnerships and resources that would be effective within this cultural and cost-context
	● Prepare educational intervention for healthcare provider referral sources
Implementation phase	● Frame the strategies and materials within local context
	● Implement cancer curricular for healthcare providers
Evaluation	● Continually improve quality via feedback loops during each stage

During the definition phase, a public health approach surveys community awareness regarding retinoblastoma, attitudes regarding cancer susceptibility and severity, and extent of blindness stigma. The definition phase necessarily investigates baseline cancer knowledge and referral trends of local health care providers [5]. The public health model then assesses available local resources and analyzes barriers to patient and provider efficacy. During the planning phase, a public health approach prioritizes partnerships and resources that would be effective within this cultural and cost-context. Development of educational resources for health care providers occurs in the planning phase. During implementation, a public health approach frames the strategies and materials utilized. Whereas success in strictly medical models is traditionally defined through calculation of inputs, outputs, and outcomes obtained at the conclusion of a project, a public health model encourage continuous quality improvement via feedback loops throughout the iterative stages of design, planning, implementation, and evaluation.

## Discussion

### Innovation of stages of change-based approach to retinoblastoma

While the innovative emphasis of this paper is to target Stages of Change models for earlier retinoblastoma diagnosis and treatment, effective intervention programs would be blind to reality to not concurrently prioritize health delivery improvements such as creation of multidisciplinary care teams, central pathology review, referral system logistics strengthening, uniform treatment protocols, reliable access to supplies and medications, cost coverage for treatment, and provision of transportation and housing. Establishment of twinning (collaboration and support partnership) programs in Guatemala and Jordan and development of integrated multidisciplinary services in Argentina and India have provided documented improvements in retinoblastoma treatment outcomes with health delivery-targeted interventions [4]. Availability of appropriate and adequate treatment is a necessary step in overcoming treatment delay.

The ability of public health interventions to impact retinoblastoma outcomes through community awareness has been well-documented [41]. A campaign linking retinoblastoma education to a national campaign in Honduras decreased median time from symptom to diagnosis from 7.2 months to 5.5 months and decreased the proportion of extraocular cases at time of presentation from 73% to 35% [42]. An early diagnosis campaign in Brazil trained public school teachers and community health workers to recognize the symptoms of retinoblastoma, resulting in decreased extraocular disease at time of presentation from 56% to <10% in less than 20 years [4]. Within the context of the Stages of Change Models, these community awareness approaches are viewed as necessary external influences, mediating the family’s ultimate readiness for treatment.

Even the most efficient of health delivery improvements and community awareness campaigns do not manifest effects on survival outcomes if the family remains undecided about seeking medical attention for leukocoria or if the family refuses enucleation. Stage-based intervention models recognize that decisions to pursue a diagnosis and engage in treatment are complex and involve a variety of motivations, knowledge levels, and readiness. Stage-based interventions target supportive behavioral strategies to help families prepare for the next decisional stage in diagnosis and treatment. Stages of Change models have been utilized effectively in breast, skin, colorectal, and cervical cancer screening decisional interventions to include settings of economic and cultural barriers [43, 44]. Although utilized in cancer screening, stage-based models have not been comprehensively applied to pediatric oncology interventions from the initial programmatic design through implementation and evaluation. A recent study describing pediatric retinoblastoma treatment completion in India documented family unwillingness to allow enucleation (20%, n = 16) as the second most common cause for treatment failure and thereby targeted the essential role of family-directed support interventions for treatment completion [39]. This study strategically timed interventions such as placing posters of patients successfully treated for retinoblastoma (with emphasis on the cosmetic outcome of prostheses) in the clinic area, creating a parent group with presence of parent experts (parents of child survivors of retinoblastoma), and intensifying family counseling sessions from time of initial clinic contact. These interventions were associated with resultant steady decline in treatment abandonment rates from 71% in 2008 to 17% in 2011 (P = 0.01) [39].

Facing system and social barriers, families should not receive blame for delay to diagnosis, delay to treatment initiation, or abandonment of treatment. At the same time, these real barriers reveal opportunities to attentively support and move families through the complex stages of readiness for diagnosis and receipt of treatment.

### Delay in time to diagnosis: method for precaution adoption process model interventions

**Figure 4:**
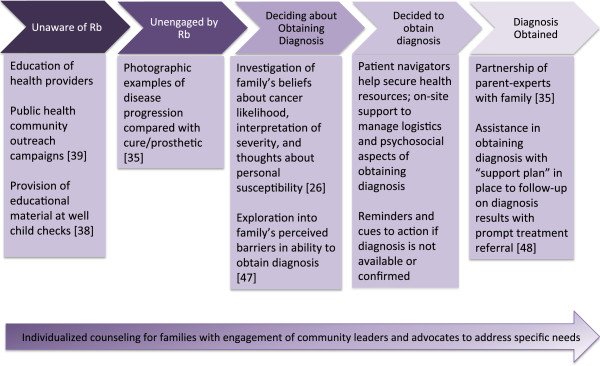
**Precaution adoption process model applied to “social interventions” for earlier diagnosis of retinoblastoma in LMIS.**

Leukocoria is the most common initial sign of retinoblastoma. Retinoblastoma remains intraocular and curable for the first 3-6 months after the first sign of leukocoria, making this a most urgent time for diagnosis before retinoblastoma spreads beyond the eye [28]. Because parents are often the first to note the ocular sign of leukocoria [45], retinoblastoma symptom awareness must prioritize family members. The Precaution Adoption Processes Model (PAPM) seeks to identify the stages involved when people commence health-protective behaviors for a dichotomous action such as seeking diagnosis for a symptom. PAPM as illustrated here presumes presence of some basic infrastructure including that to diagnosis retinoblastoma, and which in our example also included those such as well child checks, patient navigators, psychosocial support services, and prosthetic eye interventions. PAPM recognizes the factors that move people from one stage to the next, namely lack of awareness of the first symptom to the action of obtaining a diagnosis and, ultimately, the option for an individual to “exit” the model through a refusal to act (in this case, obtain a diagnosis) and terminate stage change. The barriers impeding progress toward diagnosis are targeted specifically to the decisional stage the family has reached. While many patients in truly low-income settings may present with metastatic disease beyond targeted interventions’ reach, recognition of variability in patients’ presentation within a given setting as well as promotion of efforts to address stage-specific barriers as a community can in turn galvanize the resources and social shifts necessary. Applied in a setting with appropriate resources, PAPM has potential to systematically inform development of interventions to decrease the time interval from retinoblastoma symptom onset to diagnosis by programmatically partnering with families accordingly to their expressed needs in each decisional stage leading to diagnosis (Figure 4).

### Delay in time to treatment: method for transtheoretical model interventions

**Figure 5:**
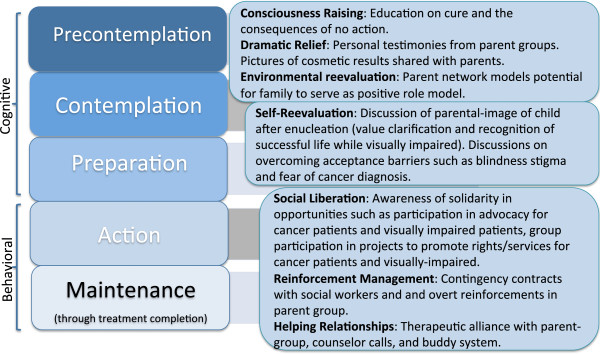
**Transtheoretical model (Linear) for retinoblastoma treatment.** The linear model represents a staged progression from precontemplation through maintenance. Maintenance in oncology care represents a starting point, as treatment completion through cure (remaining actively “maintained” through treatment) is the ultimate goal.

The Transtheoretical Model (TTM) seeks to identify the stages involved when people commence health-protective behaviors and recognizes the factors that move people from one stage to the next, ranging from Precontemplation where the family is either unaware or unengaged in making a behavioral change, to Maintenance, where Action is sustained over a period of time. The model allows for relapse and re-entry in recognizing that behavior change is complex and non-linear; additionally, the theory authors have identified processes of change that exemplify movement from one stage to another (Figure 5).

A very large proportion of patients in low-income settings present with extraocular disease, whereby decisions may involve palliation versus treatment secondary to the realities of limited resources, in which case TTM may not be readily applied for this unique context. In the particular setting where minimum resources are available (medical team and social support) and where acceptance of enucleation versus ocular salvage represents the key defining step, TTM may then be considered as an applicable theory. Using TTM, a family can be described along a series of temporal dimensions in decisional acceptance and treatment readiness with the timeline redefined in retinoblastoma due to urgency (Table 2).

**Table 2:** **Transtheoretical model’s stages of change**

Stage of change	Proposed definition
Precontemplation	Family has not started treatment and does not intend to start treatment in ___ weeks (*number of weeks locally determined*)
	● Transition emphasis: acknowledgement of urgent need
Contemplation	Family intends to start treatment but is in a “behavioral procrastination” stage without plan in place to start treatment
	● Transition emphasis: goal setting with focus on tangible plans to obtain goals
Preparation	Family has a plan of starting treatment in next days
	● Transition emphasis: Establishment of specific steps to action
Action	Family has made specific modifications to their lifestyles in preparation to start treatment
	● Transition emphasis: Community support and partnership
Maintenance	Family is actively in treatment and with intention to continue treatment
	● Transition emphasis: Assistance with problem solving and interventions in place to support family through treatment completion

Interventions at each stage primarily require creativity and commitment of planners without necessarily requiring cost-intensive resources. Examples are outlined further below, with recognition that precise applications of these social efficacy promotion efforts may vary depending on the maturity of the other health service delivery interventions and context of the local health system.

For transition from precontemplation to contemplation: This intervention engages with the family and patient with information about the urgent need to start treatment and provides personal information about the risks of not receiving treatment. As part of the consciousness-raising process, there is personalized education on cure and the consequences of no action. As part of the dramatic relief process, there is opportunity for personal testimonies from parent groups and pictures of orbital prosthesis cosmetic results shared with parents. Awareness raising interventions should continually improve upon the local quality of orbital prostheses through local partnerships with those experienced in sizing and placing orbital prosthesis with minimal infection risks. As part of environmental reevaluation, parent networks model the potential for families to envision themselves serving as positive role models for others.

For transition from contemplation to preparation: There is motivation and encouragement of the family to set goals and make specific plans through one-on-one sessions with the health team to include social workers and psychologists. As part of self-reevaluation, there are opportunities for meetings with parents of retinoblastoma survivors present to discuss the parental-image and community-image and self-image of the child after enucleation. This provides opportunity for value clarification and recognition of a successful life while visually impaired. “Family experts” (families who have a child retinoblastoma survivor member) can be available to discuss overcoming acceptance barriers such as blindness stigma and fear of cancer diagnosis.

For transition from preparation to action: The health team helps the family to create and implement specific action plans for treatment start and to set realistic goals in terms of surgical outcome, and side effects of chemotherapy or radiation therapy if needed. Team members involved in psychosocial health of the family or Child Life teams, where available, become increasingly involved to support the patient with age-appropriate medical interpretations of events (use of dolls to model what the eye bandage will look, self-expression activities, and age-appropriate coping strategies).

For transition from action to treatment: Social support and feedback are emphasized. Liberation, the belief that treatment is attainable and the commitment to act on that belief, is increasingly emphasized with family support sessions. For reinforcement management, therapeutic relationships with social workers and overt reinforcements for families to attend medical appointments may help actualize verbal commitment to treatment. This stage may introduce opportunities for group participation in projects to promote rights/services for cancer patients and visually-impaired. This transition relies on helping relationships based on therapeutic alliances whether with parent support groups, counselor telephone calls, and/or buddy systems among family networks. The maintained progression through treatment completion is an essential time to prevent abandonment of therapy. There should be continued social support, assistance with problem solving, and intervention plans in place for missed appointments or delays in care. Supporting families at this stage require creativity and resourcefulness to overcome social, transportation, educational, and financial barriers to treatment completion. This stage emphasizes reminder systems and performance-support tools, which may involve a written calendar, text messaging reminder cues for appointments, or home visits. Effective adherence data tracking under the supervision of staff trained in adherence interventions allows for monitoring and recognition of adherence.

### Family-efficacy and decisional-balance

**Figure 6:**
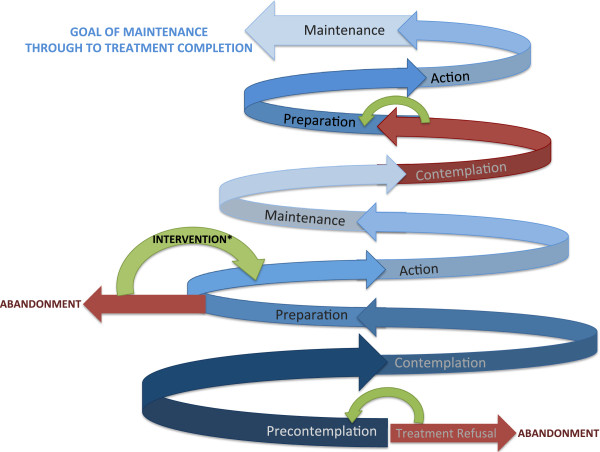
**Reality of transtheoretical model (Lived) for retinoblastoma treatment legend: The spiral model represents the lived experience as an often non-linear experience of delay to start, regressions, and recycling through stages.** The lighter the shade, the more mature the progression. Red in this model represents warning of abandonment as upfront treatment refusal or failure to complete therapy, representing regression or a recycling backward in stage progression. Blue represents a forward movement. Green represents an intervention which re-addresses family-efficacy and decisional balance to foster forward progression. Bold shades warrant additional support as patients may feel vulnerable during the newness of entrance into a stage, embarrassed by regression, or even ashamed about returning to care due to regression or recycling backward in stages.

Progression between stages of readiness for treatment initiation is not always lived out as a linear process and may involve regression to a prior stage or abandonment during a stage (Figure 6) [46]. Elements that can modulate the non-linearity of this process include family efficacy, or a family’s judgment regarding one’s ability to perform a behavior (in this case, engagement in treatment) as required to achieve a certain outcome (cure). Decisional balance, defined as the perceived benefits and perceived barriers affecting health decisions, whether financial, psychological, social, or physical, is also dynamic. The interdisciplinary care team should therefore frequently survey the family’s sense of efficacy and decisional balance to proactively intervene to promote healthy stage progression.

### Adaptation of proposed models

Each theory or model, ranging from social determinant of health models to health behavior theories to Stages of Change models, should be continually assessed and tailored for optimized application in order to achieve real-world results. The most effective approach to retinoblastoma outcomes in a particular setting will be a framework that impacts all agents: health care policies and insurance coverage, visually-impaired services in medical and social institutions, and partnerships among intra-disciplinary care teams, communities, families, and patients. The most appropriate application of theories and models will be a locally-selected, culturally-relevant, pragmatic combination of strengths from a variety of theories that best consider the multiple agents, external factors, and internal influences. The goal is for applied models to inspire and instill realistic, acceptable, measurable health improvement outcomes [47].

Each theory-based approach can bring particular strengths and potential weaknesses (examples in Table 3) and thus best practice requires adaptation and molding for disease-specific applications. Future clinical studies should consider such theory-informed analysis frameworks in addition to daily practice in contextualizing local evidence, prioritizing frameworks and data-driven actions based on the pertinent dominant stages for particular individuals and patient groups.

**Table 3:** **Stages of change approach strengths and weaknesses**

Approach	Strength	Weakness
Precaution Adaption Processes Model (PAPM)	-Dichotomous model, practical for decision-making	-“Decision not to treat” may be viewed as unacceptable
	-Incorporates a distinct unawareness stage (versus unaware OR unengaged) with opportunity for education	-Emphasis on reading materials/pamphlets may need to be locally modified to literacy rates
		-Challenge of measuring family’s exact stage of placement
Transtheoretical Model (TTM)	-Removes assumptions about immediate readiness for behavior	-Danger of evolving into a self-help model without adequate support for change when the external forces of poverty and conflicting priorities are the reason for delay
	-Recognizes different families will be in different stages	
	-Encourages inclusive, appropriately timed motivational readiness interventions	-Does not always recognize broader social and physical context
		-May unintentionally imply blame on a family, whereas much of the impetus is a fractured system of care delivery
	-Supports families between decisional stages toward acceptance	
		-Common phrases such as “self” efficacy and “self” realization may not be relevant in settings where health behaviors and outcomes are communally based
Decision to not utilize stage- based model	-Potentially streamlined decision-making	-Population characteristics, needs, and values may be overlooked when community engagement is not prioritized (available and accessible does not equal acceptable, appropriate, or equitable)
	-Time and resources centralized to making treatment available and accessible	
	-With limited funding sources, focuses resources on specific, measurable biological outcomes such as diagnostic accuracy and disease response	
		-Risk imposition of an external “evidence based approach” which is not taking local evidence and local experience into consideration to facilitate service or intervention adoption and sustainability
		-Risk suboptimal allocation and mis-prioritization of resources toward well-intentioned empiric efforts that are however poorly aligned with target populations’ current stages of readiness for change

## Summary: shared vision

“It is a terrible thing to see and have no vision”. *Helen Keller*

Retinoblastoma is often curable when diagnosed early and treated appropriately, but the prognosis is fatal when diagnosis is delayed and treatment is deferred [4]. Health service delivery-based interventions and community awareness interventions have proven helpful in decreasing lag times and improved survival outcomes, but there remains a high rate of delay in diagnosis, lag time to treatment initiation, and treatment abandonment including refusal in certain LMIS. The reality of health service and social barriers to cure obliges us to avoid placing ill-cast blame on families. Instead, a comprehensive programmatic framework that recognizes the lived context of delay provides opportunity for partnership and efficacy promotion. In striving to improve outcomes globally for curable childhood cancer conditions such as retinoblastoma, opportunity arises to include Stages of Change models for treatment completion, within the reality of lived cultural and cost contexts.

## Abbreviations

LMIS: Low- and middle- income settings
PAPM: Precaution adoption process model
Rb: Retinoblastoma
SOC: Stages of change
TTM: Transtheoretical model.
